# There’s life in the old dog yet: vitamin C as a therapeutic option in endothelial dysfunction

**DOI:** 10.1186/s13054-014-0461-9

**Published:** 2014-08-27

**Authors:** Sandra Rodemeister, Hans K Biesalski

**Affiliations:** Institute of Biological Chemistry and Nutrition, University of Hohenheim, Garbenstraße 30, 70599 Stuttgart, Germany

## Abstract

The use of vitamin C against different diseases has been controversially and emotionally discussed since Linus Pauling published his cancer studies. *In vitro* and animal studies showed promising results and explained the impact of vitamin C, particularly in cases with endothelial dysfunction. Indeed, studies (reviewed in this issue of *Critical Care* by Oudemans-van Straaten and colleagues) using high-dose vitamin C and the parenteral route of application seem to be more successful than oral vitamin C delivery.

Endothelial dysfunction (ED) – as a pathology emerging, for example, out of surgery-elicited ischemia–reperfusion injury or arising during sepsis – contributes to tissue injury, thereby promoting the development of multiple organ failure [1] and as a consequence elevating the length of hospital stay and costs. To deal with this challenging situation, antioxidant therapy – in particular, the use of vitamin C – has been frequently recommended and is under continuous controversial discussion. In this issue of *Critical Care*, Oudemans-van Straaten and colleagues give an overview of the current experimental and clinical data for vitamin C in this context [2].

The pathophysiological situation of ED – including impaired regulation of vascular tone, compromised endothelial barrier function and loss of the endothelium’s antithrombotic and antiatherogenic properties [3] – is mainly caused by two highly connected mechanisms: the loss of nitric oxide (NO) availability and function; and the highly increased production of reactive oxygen species, especially superoxide and peroxynitrite. Both of these mechanisms result from an uncoupling of endothelial nitric oxide synthase (eNOS) that occurs when tetrahydrobiopterin, a cofactor of eNOS, is not sufficiently available. In this uncoupled state, eNOS produces superoxide instead of NO (for a comprehensive review, see [4]). Furthermore, the accompanying inflammation leads to the activation of NADPH oxidase and inducible NO synthase, producing high amounts of superoxide and NO, respectively, thereby further promoting the formation of peroxynitrite.

Vitamin C can ameliorate this situation by different methods of action (reviewed in detail in [2]). First, vitamin C inhibits the activation of NADPH oxidase and inducible NO synthase, as shown both *in vitro* and in animal models, thereby preventing the formation of reactive oxygen species. Furthermore, as vitamin C is necessary for the reductive recycling of tetrahydrobiopterin, it counteracts the uncoupling of eNOS, thereby contributing to the recovery of endothelial function.

Interestingly, although there is consensus concerning the approach of reversing uncoupled eNOS via provision of tetrahydrobiopterin to combat ED [4,5], vitamin C seems not to be considered as a therapeutic option. This might be due to some contradictory results concerning the impact of vitamin C in the setting of vascular oxidative stress, ranging from beneficial effects in small clinical studies (reviewed in [2]) to no effect in a large-scale randomized clinical trial [6]. This latter result might have given the temporary deathblow to vitamin C for ED therapy.

However, high-dose vitamin C administration – a phrase frequently occurring in the review [2] – cannot be achieved with oral application. Indeed, as Padayatty and coworkers showed in their investigation of vitamin C kinetics, most notably it is the route of vitamin C administration that has to be taken into account [7]. As the intestinal absorption for vitamin C is rapidly saturated, high dosages of vitamin C (>500 mg) have to be applied intravenously and not via the oral route, as practiced [6]. The failure of that study to show an effect is simply that the investigators chose the wrong route of application.

In fact, there are two reasons to administer vitamin C parenterally: prevention or treatment of ED (as discussed in [2]), and compensation of a vitamin C deficit following surgery.

Indeed, the results of several clinical investigations in patients undergoing cardiac surgery with use of cardiopulmonary bypass – the latter necessarily evoking ischemia–reperfusion injury – showed a strong and long-lasting decrease in vitamin C plasma concentrations following surgery [8,9]. Furthermore, Cowley and coworkers demonstrated a positive relationship between plasma antioxidant potential and survival rate in patients with severe sepsis [10]. Supplementation of vitamin C in patients undergoing any surgery eliciting ischemia–reperfusion injury or suffering sepsis therefore seems to be highly recommendable.

For reconstitution of adequate vitamin C plasma levels in critically ill patients, high doses of vitamin C (3 g/day) given intravenously for 3 days or more are needed [11]. An argument against the high-dose application was the risk of an increased formation of vitamin C radicals. However, in a study with healthy volunteers we showed that parenteral vitamin C (750 or 7,500 mg for 6 consecutive days) does not lead to any pro-oxidative effects [12].

We need to consider that intravenous application of vitamin C has nothing to do with the known physiology of vitamin C from nutrition or oral supplements. Circumventing the physiological control of vitamin C following oral intake may lead to very different effects that may be distinct from those we know from nutrition studies. Parenteral application is a pharmacological approach. Indeed, little is known about the distribution, metabolism, action and degradation as well as the optimal moment and time course for intravenous high-dose supplementation of vitamin C in the context of ED.

Controlled studies are needed to elucidate whether early high-dose administration of vitamin C might help to keep the plasma level in a normal range and to prevent ED.

## Abbreviations

ED: Endothelial dysfunction
eNOS: Endothelial nitric oxide synthase
NO: Nitric oxide
